# Temperature and blood flow distribution in the human leg during passive heat stress

**DOI:** 10.1152/japplphysiol.00965.2015

**Published:** 2016-01-28

**Authors:** Scott T. Chiesa, Steven J. Trangmar, José González-Alonso

**Affiliations:** Centre for Human Performance, Exercise and Rehabilitation, Brunel University London, Uxbridge, United Kingdom

**Keywords:** leg blood flow, heat stress, hemodynamics

## Abstract

The influence of temperature on the hemodynamic adjustments to direct passive heat stress within the leg's major arterial and venous vessels and compartments remains unclear. Fifteen healthy young males were tested during exposure to either passive whole body heat stress to levels approaching thermal tolerance [core temperature (T_c_) + 2°C; *study 1*; *n* = 8] or single leg heat stress (T_c_ + 0°C; *study 2*; *n* = 7). Whole body heat stress increased perfusion and decreased oscillatory shear index in relation to the rise in leg temperature (T_leg_) in all three major arteries supplying the leg, plateauing in the common and superficial femoral arteries before reaching severe heat stress levels. Isolated leg heat stress increased arterial blood flows and shear patterns to a level similar to that obtained during moderate core hyperthermia (T_c_ + 1°C). Despite modest increases in great saphenous venous (GSV) blood flow (0.2 l/min), the deep venous system accounted for the majority of returning flow (common femoral vein 0.7 l/min) during intense to severe levels of heat stress. Rapid cooling of a single leg during severe whole body heat stress resulted in an equivalent blood flow reduction in the major artery supplying the thigh deep tissues only, suggesting central temperature-sensitive mechanisms contribute to skin blood flow alone. These findings further our knowledge of leg hemodynamic responses during direct heat stress and provide evidence of potentially beneficial vascular alterations during isolated limb heat stress that are equivalent to those experienced during exposure to moderate levels of whole body hyperthermia.

## Abstract (precis)

The ability of direct heat stress to increase limb blood flow is well known, but the magnitude and profile of hemodynamic responses within the major vessels of the leg have not been explored. Here, we systematically characterize these responses through a wide range of heat stress levels and show that isolated leg heating confers potentially beneficial hemodynamic changes equivalent to those of moderate whole body hyperthermia, with these hemodynamic adjustments being predominantly driven by local temperature-sensitive mechanisms.

## NEW & NOTEWORTHY

*The ability of direct heat stress to increase limb blood flow is well known, but the magnitude and profile of hemodynamic responses within the major vessels of the leg have not been explored. Here, we systematically characterize these responses through a wide range of heat stress levels and show that isolated leg heating confers potentially beneficial hemodynamic changes equivalent to those of moderate whole body hyperthermia, with these hemodynamic adjustments being predominantly driven by local temperature-sensitive mechanisms*.

upon exposure to high environmental temperatures, numerous cardiovascular adjustments result in significant increases in blood flow to the skin and extremities in conjunction with decreases in blood flow to the major thoracic vascular beds (24, 34, 36). The factors controlling these changes and the distribution of blood flow to different tissues within the limbs, however, are still not fully characterized and understood. A significant proportion of peripheral tissues (∼25%) within the human body are contained within the legs, and substantial vasodilation within these vascular beds have been postulated to contribute to certain known detrimental conditions of heat stress, such as the increased cardiac strain or incidence of orthostatic intolerance commonly reported in hyperthermic individuals. In contrast, however, this same vasodilatory response may possess the potential, when appropriately applied, to confer benefits to vascular function through increases in tissue blood flow and shear rates alongside decreases in proatherogenic oscillatory flow patterns within the major vessels. Although the hemodynamic response to heat stress has previously been characterized in the human forearm (6, 25, 27), the presence of a much greater incidence of vascular dysfunction in the lower limbs (20) suggests that lower limb hemodynamic responses to heat stress may differ, both in the leg as a whole and in the individual vessels perfusing the major vascular beds.

Recent work from our laboratory has shown that increases in blood flow to the human leg during exposure to both isolated leg heating and moderate levels of direct whole body passive heat stress are mediated solely through changes in local vascular conductance and that these changes are closely associated with local temperature, suggesting a dominant role of local temperature-sensitive mechanisms during this direct form of whole body heat stress (7). Although this study has investigated the role of temperature on leg blood flow distribution during moderate levels of heat stress, no study to date has assessed the effect of local vs. central temperature contributions during more severe levels of whole body heat stress. These responses will be important if the use of heat stress as a therapeutic intervention is to be further investigated in patient populations and ideally optimized to provide maximal vascular benefit alongside minimal detrimental heat-stress-associated conditions. Despite the leg hyperemic response to heat stress previously being documented in a number of studies (7, 13, 29), the relative distribution of blood flow within not only the leg itself (i.e., thigh vs. lower leg), but also the individual major vessels supplying and draining these tissues, remains relatively unexplored. While the GSV is often claimed to be the major drainage vessel of the skin circulation, relatively little is known of the contribution of the deep veins to venous return during heat stress, with only one study to date investigating this phenomenon and only to heat stress approaching moderate levels (1). In addition, no study to date has characterized blood flow profiles within the major vessels supplying the leg, which may have the potential to enhance anti-atherogenic shear responses similar to those shown in the human forearm, or compared the effectiveness of local heating interventions vs. whole body heat stress in eliciting these responses.

This study, therefore, aimed to *1*) comprehensively investigate blood flow distribution and profiles within the major vessels and compartments (thigh and lower leg) of the human leg during progressive elevations in heat stress to levels approaching that of an individual's thermal tolerance, and *2*) build on previous work during moderate heat stress by investigating the relative contribution of local and central temperature in the distribution of leg tissue perfusion at higher levels of heat stress. We hypothesized that *1*) direct passive heat stress would lead to progressive elevations in blood flow to all vessels of the leg alongside corresponding beneficial changes in flow profiles within the vessels themselves, and 2) that local control mechanisms would continue to be the dominant controlling mechanism responsible for these perfusion and hemodynamic changes during the direct heating.

## METHODS

### Ethical Approval

Informed written consent was obtained from each participant before commencing the study. All procedures were approved by the Brunel University London Research Ethics Committee (RE27-13) and conformed to the *Declaration of Helsinki*.

### Participants

Fifteen healthy males (age: 23 ± 5 yr; height: 181 ± 7 cm; and weight: 75 ± 12 kg) were recruited to participate in two separate studies, with each participant exposed to either passive whole body heat stress (*study 1*; *n* = 8) or, in a follow-up study, isolated single leg heat stress (*study 2*; *n* = 7). Participants abstained from alcohol, caffeine, and strenuous exercise in the 24 h leading up to the day of testing.

### Experimental Protocols

**Fig. 1.**
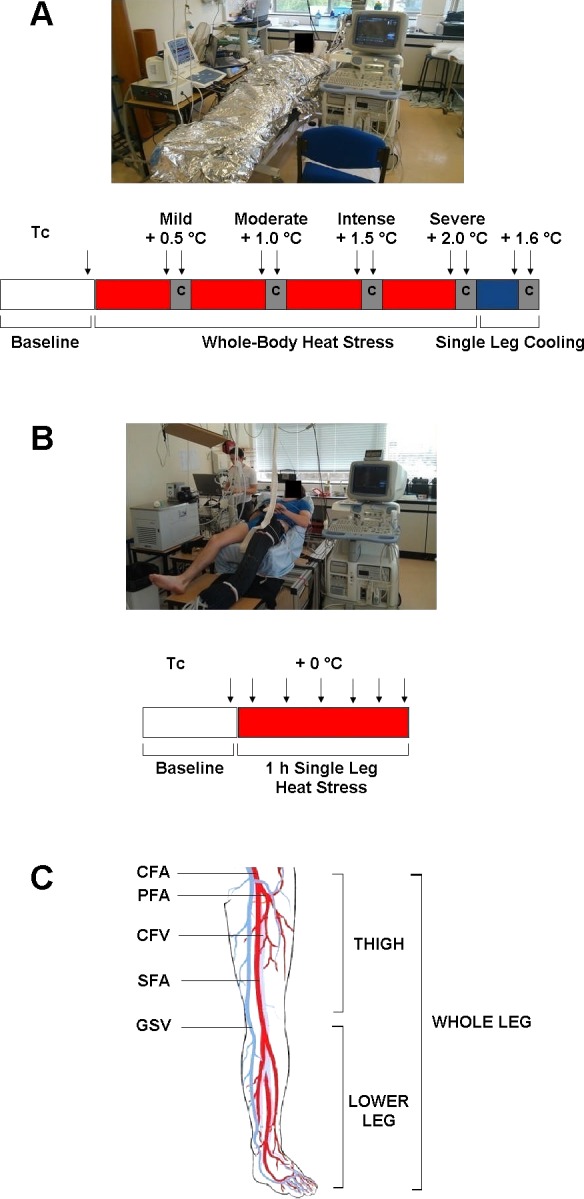
Sequence of the experimental protocols and schematic of the leg major vessels and anatomical sections. *A*: in *study 1*, following baseline measurements, participants were heated to core temperature (T_c_) + 2°C with measurements (denoted by arrows) taken during mild (+0.5°C), moderate (+1°C), intense (+1.5°C), and severe (+2°C) heat stress, both before and after arterial cuff occlusions (denoted by C) at the level of the knee. Following the whole body heat stress protocol, the left leg was rapidly cooled and further measurements taken as before. *B*: participants in *study 2* rested in ambient thermoneutral conditions while a single leg was heated for a duration of 1 h. *C*: illustration of the leg's major supplying [common, superficial, and profunda femoral arteries (CFA, SFA, and PFA)] and draining vessels [common femoral vein (CFV) and great saphenous vein (GSV)] and the anatomical differentiation between the thigh and the lower leg.

Experimental setups for both studies are depicted in Fig. 1. In *study 1*, each participant visited the laboratory on two occasions separated by at least 1 wk. These consisted of a shortened familiarization trial in the initial visit to estimate whole body sweat rate, followed by the main experimental trial at least 1 wk later. In the familiarization trial, participants attended the laboratory at 9 AM following ingestion of their usual breakfast and were weighed and measured in a seminude state (SECA 798). Following instrumentation, participants were fitted with a custom-built water-perfused suit designed to cover the entire body, before being wrapped in survival blankets to prevent heat loss to the surrounding environment and left to rest in the supine position for the remainder of the study. The suit was connected to a thermostatically controlled water circulator (Julabo F-34; Seelbach) to allow the constant perfusion of 50°C water and subsequent manipulation of body temperature, and participants were passively heated until core temperature (T_c_) increased by 1°C. Following the end of the intervention, participants were weighed once again and whole body sweat rate was estimated in liters per hour as (preheating mass in kilograms − postheating mass in kilograms)/duration of heating in hours.

The experimental trial consisted of a similar protocol to the familiarization trial but with participants exposed to a more severe level of heat stress than their initial visit. Full body passive heating was continued until participants reached their level of thermal tolerance or achieved a T_c_ of at least 2°C above baseline. Levels of heat stress were defined as mild (+0.5°C), moderate (+1°C), intense (+1.5°C), and severe (+2°C). Thermal tolerance was defined by participants displaying symptoms of presyncope, reporting symptoms of nausea, or indicating they were no longer willing to continue. Participants were provided with water every 20 min at a rate equal to their calculated sweat rate in the familiarization trial and at a temperature approximately equal to that of T_c_. At ∼20-min intervals throughout the heating protocol, complete arterial occlusion at the level of the knee was performed to assess blood flow distribution to the thigh and the lower leg, with the thigh defined as the section of the leg between the hip and the knee and the lower leg defined as the section below the knee. Occlusions were carried out for 5 min via the inflation of a pneumatic cuff at the level of the knee (immediately distal to the patella) to 240 mmHg (Hokanson E20 Rapid Cuff Inflator and CC17 Cuff, Bellevue, WA). A minimum of 20 min always elapsed between the deflation of the pneumatic cuff and the next measurement of whole leg blood flow to ensure that no residual effects of the postreactive hyperemia distorted the next set of measurements. Following the attainment of the target T_c_, one leg of the water-perfused suit was removed and the exposed limb was completely surrounded in bags of crushed ice for 10 min to facilitate rapid skin and subcutaneous tissue cooling. This intervention resulted in the presence of a low local temperature/high core temperature state, allowing the contribution of each of these regulatory inputs to flow distribution to be assessed. Following measurements of blood flow in the different vessels after 5 min of local cooling, an arterial occlusion was performed at the level of the knee using the pneumatic cuff and temperature and hemodynamic responses were measured at various depths within the cooled thigh to establish the relationships between local and systemic temperature and blood flow within the deep and superficial tissues.

In *study 2*, participants reported to the laboratory at a similar time of day and, following instrumentation, rested in the supine position while a single leg was fitted with one leg of the water-perfused suit, designed to cover the entire limb. Similar to *study 1*, the single water-perfused trouser leg was subsequently perfused with 50°C water to raise limb tissue temperatures in the isolated leg alone. Blood flow and temperature measurements were recorded every 10 min in the experimental limb for a duration of 1 h, with contralateral control leg blood flow recorded at 0, 30, and 60 min.

### Temperature Measurements

T_c_ was measured using an esophageal thermistor (PhysiTemp, Clifton, NJ). Following application of local anesthetic gel (1% lidocaine) to the dominant nostril, the probe was inserted into the nasal cavity and advanced to a level roughly one-fourth that of standing height to sit behind the left atrium (22). Skin temperature (T_sk_) was measured using wireless telemetry buttons (iButtons; Maxim) fitted to the arm, chest, thigh, and calf; and mean whole body skin temperature (T̄_sk_) was calculated using a standard weighted formula (31). Mean leg skin temperature was calculated as the average of thigh and calf measurements. Muscle (T_m_) and subcutaneous (T_sc_) temperatures were measured using sterile implantable thermocouples inserted into the mid-portion of the vastus lateralis muscle using a 22-gauge catheter. Local anesthetic gel (1% lidocaine) was applied to the site of measurement before insertion to minimize any discomfort. T_m_ was measured at a depth of ∼2-3 cm, while T_sc_ was measured directly under the skin. Mean leg temperature (T̄_leg_) was calculated as a ratio of leg T_sk_, T_sc_, and T_m_, weighted by the volume of each of these tissue compartments in the leg (43) and averaged to provide an indication of mean tissue temperature across the leg. The lack of T_sc_ measurements during single leg heating resulted in the calculation of T̄_leg_ as a ratio of T_sk_ and T_m_ alone. The close tracking of T_sk_ and T_sc_ during whole body heat stress, however, suggested that results between the two studies would be comparable despite this limitation.

### Hemodynamic Measurements

Arterial blood pressure and heart rate were measured noninvasively by infrared photoplethysmography (Finometer; FMS) via a cuff on the middle finger of the right hand. Stroke volume was estimated using the ModelFlow method included with the Beatscope computer software package (Beatscope; FMS), with cardiac output calculated as stroke volume × heart rate following corrections for age, height, and weight (44). Blood flow was repeatedly measured during the different stages of heat stress in the common, superficial, and profunda femoral arteries (CFA, SFA, and PFA, respectively) and the GSV using a duplex Doppler ultrasound system (Vivid 7 Dimension; GE Medical, Horton, Norway) with a 10-MHz linear array transducer probe (GE Medical Systems). Common femoral vein (CFV) blood flow was calculated as CFA blood flow minus GSV blood flow. Custom-made flaps in the water-perfused suit and surrounding survival blankets allowed the probe to be placed against the skin with minimal heat loss to the surrounding environment, while a small gap between ice packs allowed the same measurements to be taken during subsequent isolated leg cooling. All arterial measurements were taken at a distance of at least 2 cm from the bifurcation into the SFA and PFA to minimize disruption to measurements due to turbulent flow, whereas flow through the GSV was measured immediately distal to the preterminal valve separating the vein from the saphenofemoral junction in an attempt to prevent influx of flow from accessory veins draining areas other than the leg. Measurements of GSV blood flow using Doppler ultrasound have previously been shown in a number of studies to be reliable and repeatable (1, 26). Blood flow through each vessel was calculated as the product of the average vessel cross-sectional area obtained from three two-dimensional B-mode images and the mean velocity averaged over two 16-s Doppler scans (32 s total). Arterial diameter was consistently measured at peak systole (30), identified by an overlaid ECG trace, while venous diameter was measured every 2 s over the duration of one velocity trace (16 s total) to account for diameter changes due to both cardiac and respiratory cycles. Arterial and venous blood flows (BF) were calculated in ml/min using the equation BF = *V*_mean_ × π × (*D*/2)^2^ × 60, where *V*_mean_ is the time-averaged mean velocity of the blood expressed as cm/s, π is a mathematical constant, *D* is the diameter of the vessel in cm, and 60 is a constant employed to convert the units to ml/min. Shear rates were calculated using the formula SR = (4 × *V*_mean_)/*D*, where SR is shear rate; *V*_mean_ is mean, anterograde, or retrograde blood velocity; and *D* is vessel diameter. Oscillatory shear index was calculated using the equation OSI = (SR_ret_)/(SR_ant_ + SR_ret_), where OSI is oscillatory shear index, SR_ret_ is mean retrograde shear rate, and SR_ant_ is mean anterograde shear rate. The OSI calculation provides a dimensionless variable that indicates the relative contribution of anterograde and retrograde shear profiles over the duration of a cardiac cycle and ranges from 0 (pure anterograde shear) to 0.5 (pure oscillatory shear).

### Statistical Analysis

A one-way repeated-measures ANOVA was used to test for differences over time, with Holm-Bonferroni post hoc testing employed to identify the time points at which changes occurred once a significant effect was found. Multiple regression for within-subject repeated measures was used for the analysis of the relationship between leg blood flow and T̄_leg_ (4). Differences in OSI between whole body and isolated lower-limb heat stress were compared using an independent samples *t*-test. All statistical analyses were carried out using SPSS (Version 20; IBM, Armonk, NY) with results expressed as mean ± SE. Significance was set at *P* < 0.05.

## RESULTS

### Temperature Responses During Whole Body and Isolated Leg Heat Stress

**Fig. 2.**
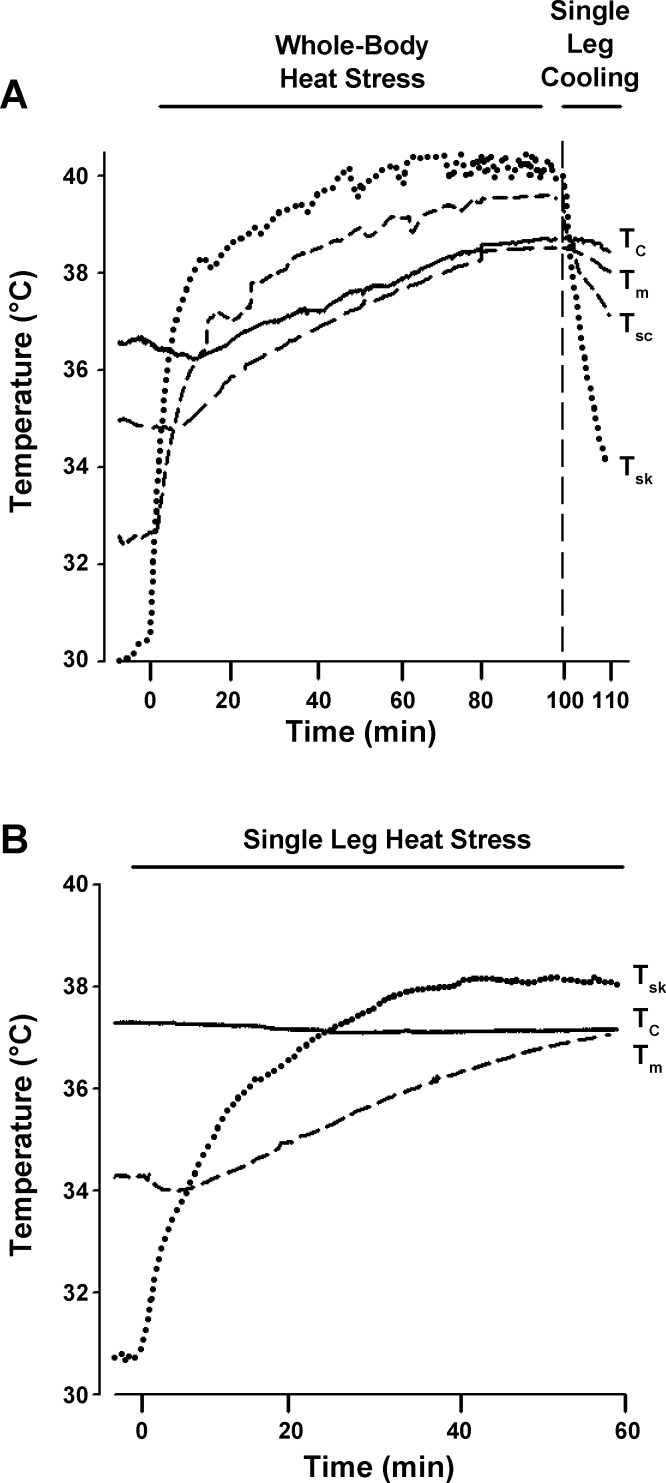
Temperature responses to passive whole body heat stress followed by single leg cooling and single leg heat stress. *A*: representative trace from a single participant showing typical core, muscle, subcutaneous, and skin temperature responses during progressive whole body heat stress followed by single leg cooling (indicated by dashed vertical line) (*study 1*). *B*: representative trace from a single participant showing typical core, muscle, and skin temperature responses during single leg heat stress (*study 2*).

Full temperature responses can be seen in Table 1, while representative responses from one subject during both whole body and isolated leg heat stress are displayed in Fig. 2. In *study 1*, passive whole body heat-stress increased T̄_sk_ and T_sc_ rapidly upon commencement of heat stress before leveling off following 40–50 min and remaining elevated for the remainder of the experiment (final values ∼6°C higher than baseline for both; *P* < 0.05 for all; Fig. 2*A*). As determined by the experimental design, T_c_ increased progressively up to 2.0 ± 0.1°C (mean heating duration: 94 ± 5 min) while T_m_ increased in a linear fashion from 34.9 ± 0.3 to 38.3 ± 0.1°C. Subsequent cooling of a single leg for 10 min reduced leg T̄_sk_, T_sc_, and T_m_ by 11.4, 7.5, and 1.5°C, respectively, such that T̄_sk_ and T_sc_ tended to be reduced below baseline values while T_m_ and T_c_ remained elevated.

**Table 1.** Temperature and hemodynamic responses to whole body heat stress followed by single leg cooling and isolated single leg heat stress

	Whole Body Heat Stress						Isolated Single Leg Heat Stress	
	Baseline	Mild	Moderate	Intense	Severe	Leg cooling	Baseline	End
T_c_, °C	36.8 ± 0.1	37.2 ± 0.1*	37.8 ± 0.1*	38.3 ± 0.1*	38.8 ± 0.1*	38.4 ± 0.1†	37.2 ± 0.4	37.2 ± 0.6
T̄_sk_, °C	33.1 ± 0.4	37.5 ± 0.3*	38.4 ± 0.2*	39.0 ± 0.2*	39.4 ± 0.2*	38.2 ± 0.2†	31.1 ± 0.4	38.9 ± 0.6*
T_sc_, °C	33.5 ± 0.6	37.0 ± 0.1*	37.8 ± 0.3*	38.2 ± 0.2*	39.0 ± 0.4*	31.5 ± 1.2†	—	—
T_m_, °C	34.9 ± 0.3	36.2 ± 0.2*	37.0 ± 0.2*	37.6 ± 0.2*	38.3 ± 0.1*	36.8 ± 0.2†	34.5 ± 0.5	36.8 ± 0.1*
T_thigh_, °C	33.4 ± 0.9	40.3 ± 0.9*	40.2 ± 0.7	40.1 ± 0.3	40.5 ± 0.3	29.0 ± 5.1†	31.3 ± 0.2	39.1 ± 0.5
T_calf_, °C	31.5 ± 0.3	33.8 ± 0.5*	35.9 ± 0.3*	37.4 ± 0.2*	37.8 ± 0.2	26.9 ± 5.3†	30.7 ± 0.3	38.3 ± 0.5
T̄_leg_, °C	34.4 ± 0.4	35.9 ± 0.3	37.0 ± 0.1	37.8 ± 0.1	38.7 ± 0.3	36.2 ± 0.3	34.0 ± 1.3	37.1 ± 0.3
CFA, l/min								
Whole leg	0.31 ± 0.03	0.53 ± 0.07*	0.86 ± 0.07*	1.24 ± 0.10*	1.22 ± 0.11	0.98 ± 0.04†	0.25 ± 0.02	0.76 ± 0.08*
Leg occlusion	0.22 ± 0.02	0.44 ± 0.02*	0.64 ± 0.05*	0.65 ± 0.05	0.71 ± 0.04	0.55 ± 0.05†	—	—
SFA, l/min								
Whole leg	0.17 ± 0.01	0.41 ± 0.05*	0.73 ± 0.08*	0.91 ± 0.08*	0.95 ± 0.09	0.76 ± 0.06†	0.13 ± 0.01	0.46 ± 0.07*
Leg occlusion	0.11 ± 0.02	0.28 ± 0.03*	0.39 ± 0.04*	0.42 ± 0.04	0.43 ± 0.04	0.29 ± 0.05†	—	—
PFA, l/min								
Whole leg	0.10 ± 0.02	0.12 ± 0.02*	0.18 ± 0.02*	0.22 ± 0.04*	0.26 ± 0.04*	0.19 ± 0.02†	0.08 ± 0.01	0.22 ± 0.05*
Leg occlusion	0.08 ± 0.02	0.16 ± 0.02*	0.20 ± 0.03*	0.24 ± 0.03*	0.27 ± 0.03*	0.17 ± 0.02†	—	—
CFV, l/min								
Whole leg	0.28 ± 0.03	0.40 ± 0.05*	0.66 ± 0.07*	0.98 ± 0.10*	0.97 ± 0.10	0.78 ± 0.03†	—	—
Leg occlusion	0.20 ± 0.02	0.34 ± 0.02*	0.49 ± 0.04*	0.49 ± 0.04	0.54 ± 0.03	0.40 ± 0.04†	—	—
GSV, l/min								
Whole leg	0.02 ± 0.01	0.13 ± 0.02*	0.21 ± 0.02*	0.25 ± 0.02*	0.25 ± 0.03	0.22 ± 0.05	—	—
Leg occlusion	0.01 ± 0.01	0.10 ± 0.01*	0.15 ± 0.01*	0.16 ± 0.02	0.17 ± 0.02	0.15 ± 0.03	—	—
Q̇, l/min	6.4 ± 0.5	7.3 ± 0.3*	8.5 ± 0.6*	9.3 ± 0.6*	9.7 ± 0.6*	8.4 ± 0.5†	6.2 ± 0.5	6.4 ± 0.5
HR, beats/min	60 ± 3	72 ± 3*	85 ± 4*	96 ± 4*	106 ± 3*	93 ± 3†	65 ± 8	72 ± 4
SV, ml	107 ± 8	102 ± 8	100 ± 8*	98 ± 9	91 ± 7*	92 ± 7	95 ± 5	90 ± 7
MAP, mmHg	91 ± 3	78 ± 2*	80 ± 2	81 ± 2	82 ± 3	80 ± 2	78 ± 3	81 ± 3

Values are means ± SE for 8 participants (*study 1*) and 7 participants (*study 2*). T_c_, core temperature; T_m_, leg muscle temperature; T_sc_, leg subcutaneous temperature; T̄_sk_, mean leg skin temperature; T_thigh_, thigh skin temperature; T_calf_ calf skin temperature; T̄_leg_ mean leg temperature; CFA, common femoral artery; SFA, superficial femoral artery; PFA, profunda femoral artery; CFV, common femoral vein; GSV, great saphenous vein; Q̇, cardiac output; HR, heart rate; SV, stroke volume; MAP, mean arterial pressure.

* *P* < 0.05, significantly different from previous condition.

† *P* < 0.05, significantly different from severe whole body heat stress.

In *study 2*, isolated leg heat stress led to increases in leg T̄_sk_ and T_m_ from 31.1 ± 0.4 to 38.9 ± 0.6°C and from 34.5 ± 0.5 to 36.8 ± 0.1°C, respectively (*P* < 0.05 for both), with T_c_ remaining unchanged throughout. The time course of the temperature responses of a representative participant during isolated leg heat stress is illustrated in Fig. 2*B*.

### Leg Perfusion and Systemic Hemodynamic Responses to Whole Body Heat Stress, Lower Leg Occlusion, and Single Leg Cooling

#### Passive whole body heat stress.

**Fig. 3.**
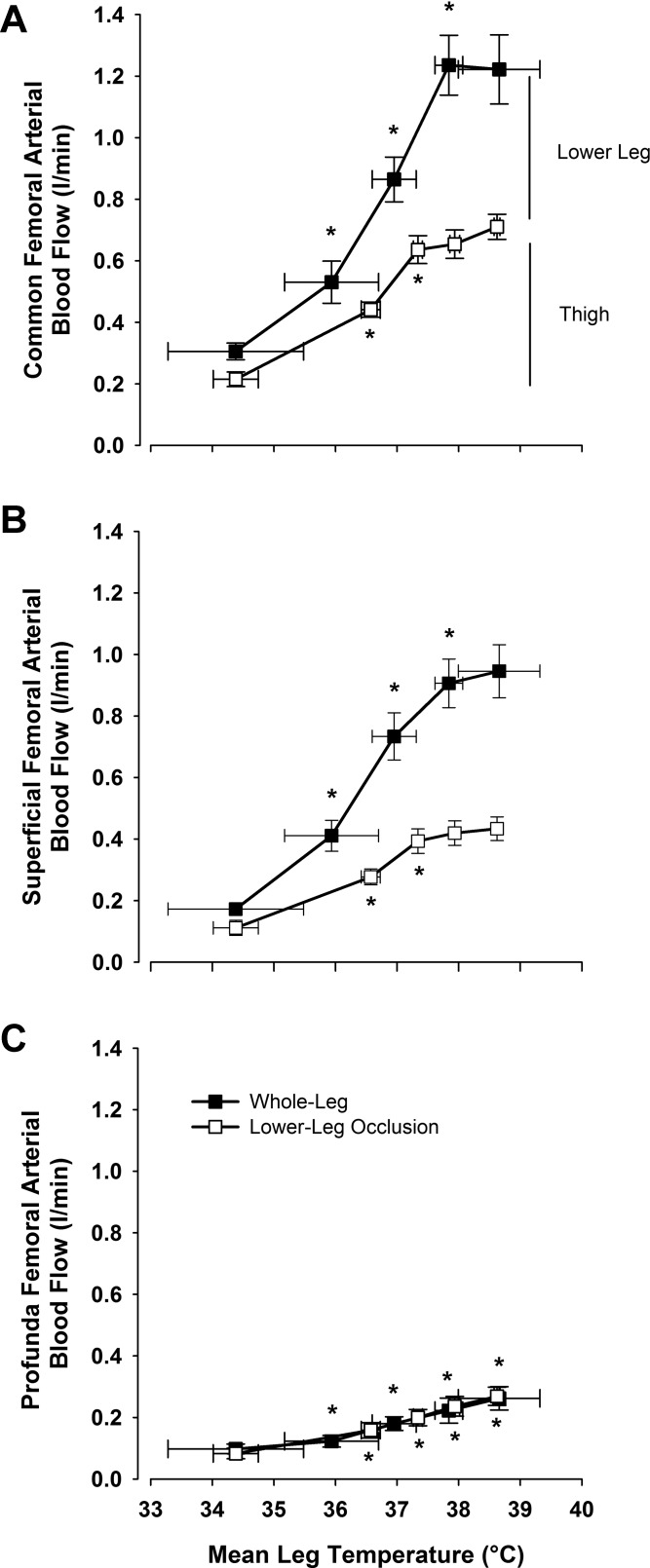
Leg blood flow distribution in the thigh and leg during passive whole body heat stress and leg occlusion. Blood flow distribution to each of the major conduit arteries during passive heat stress. Thigh blood flow is represented by CFA blood flow following a cuff occlusion at the level of the knee, whereas lower leg blood flow is the difference between thigh blood flow and whole leg blood flow. *A*: CFA. *B*: SFA. *C*: PFA. Data are mean ± SE for 8 subjects. *P < 0.05, significantly higher than previous condition.

**Fig. 4.**
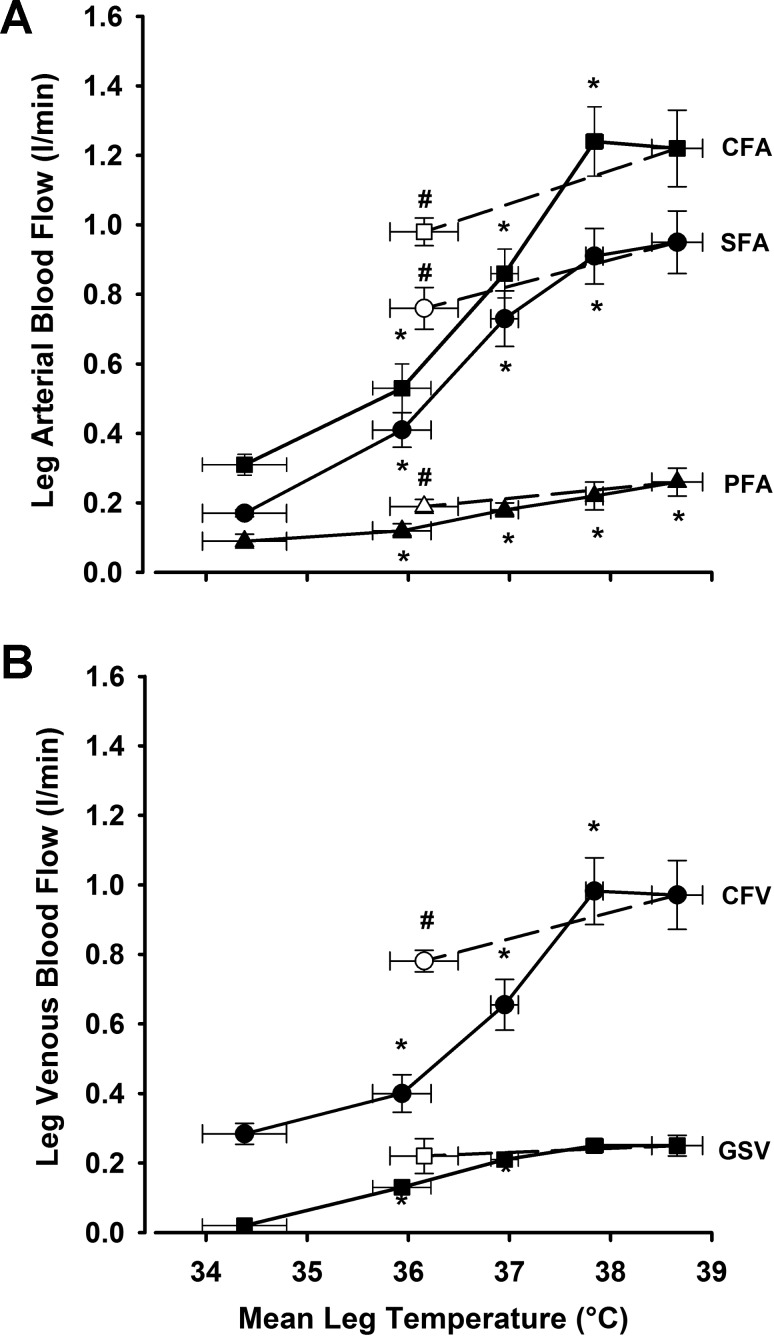
Relationship between leg hemodynamic responses and mean leg temperature during passive whole body heat stress followed by single leg cooling. CFA, SFA, and PFA blood flows vs. mean leg temperature (*A*) and GSV and CFV blood flow (*B*) during passive heat stress and rapid single leg cooling. Data are mean ± SE for 8 subjects. *Significantly higher than previous condition. #*P* < 0.05, significantly lower than severe heat stress.

In *study 1*, CFA blood flow, an index of whole leg blood flow, increased in a linear fashion from 0.30 ± 0.03 l/min at baseline to 1.24 ± 0.10 l/min during intense heat stress (+0.93 l/min) but showed no further increases upon progression to severe heat stress (final values: 1.22 ± 0.11 l/min; Table 1 and Fig. 3*A*). This plateau occurred despite further elevations in both T_c_ and T̄_leg_. SFA blood flow showed a similar response (+0.73 l/min), with an apparent plateau during severe heat stress (Fig. 3*B*), while PFA flow displayed a modest yet steady increase over the entire duration of passive heat stress (+0.17 l/min; Fig. 3*C*). CFV and GSV blood flow showed similar responses to that observed on the arterial side of the circulation, with increases from baseline to intense heat stress (+0.80 and +0.23 l/min, respectively; *P* < 0.05), before plateauing during severe heat stress (Fig. 4). At the systemic level, cardiac output (Q̇) increased from 6.4 ± 0.5 l/min at baseline to 9.7 ± 0.7 l/min during severe heat stress. Mean arterial pressure (MAP) decreased from baseline to moderate heat stress (90 ± 3 to 80 ± 2 mmHg; *P* < 0.05) before plateauing for the remainder of heating (final value: 82 ± 3 mmHg).

#### Lower leg occlusion.

Lower leg occlusion at the level of the knee allowed blood flow distribution to the thigh and lower leg to be determined (Fig. 3). Thigh blood flow increased with increasing T_c_ and T̄_leg_ temperatures from baseline to levels of moderate heat stress (0.22 ± 0.02 to 0.64 ± 0.05 l/min; *P* < 0.05), leveling off for the remainder of the protocol despite further increases in T_c_ and T̄_leg_ of ∼1 and 2°C, respectively (*P* < 0.05; Table 1 and Fig. 3*A*). In contrast, lower leg blood flow showed significant increases until intense levels of heat stress were obtained, before also leveling off at 0.5 l/min before severe heat exposure. Blood flow to the lower leg was supplied exclusively by the SFA (Fig. 3*B*), as PFA blood flow remained unaffected by lower leg occlusion at all levels of heat stress (Fig. 3*C*).

#### Isolated leg cooling.

Single leg cooling following severe heat stress, both with and without leg occlusion, led to a significant decrease in blood flow to the CFA, SFA, PFA, and CFV (10–20%; *P* < 0.05; Fig. 4) but not the GSV (*P* = 0.21). These decreases in limb blood flow occurred alongside decreases in heart rate (106 ± 3 to 93 ± 3 beats/min; *P* < 0.05) and a subsequent decrease in Q̇ (9.7 ± 0.6 to 8.4 ± 0.5 l/min; *P* < 0.05), as stroke volume remained unchanged.

### Leg Perfusion and Systemic Hemodynamic Responses During Isolated Leg Heat Stress

Isolated leg heat stress in *study 2* led to significant increases in blood flow through the CFA (0.25 ± 0.02 to 0.76 ± 0.08 l/min; 3-fold increase), SFA (0.13 ± 0.01 to 0.46 ± 0.07 l/min; 3.5-fold increase), and PFA (0.08 ± 0.01 to 0.22 ± 0.05 l/min; 2.5-fold increase; *P* < 0.05 for all). In the contralateral control limb, no significant changes in flow were observed during either the cooling or heating protocol (*P* > 0.05). At the systemic level, there were no significant changes in hemodynamic variables during either the cooling or heating protocol.

### Flow Profile and Shear Rate Responses During Whole Body and Isolated Leg Heat Stress

**Fig. 5.**
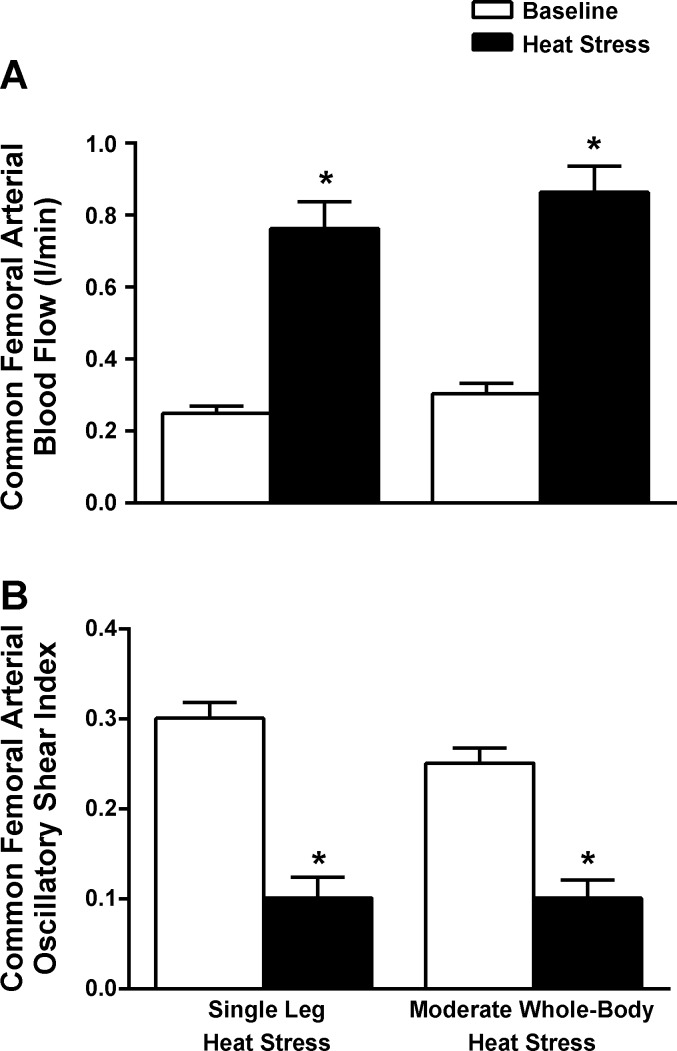
CFA blood flow and oscillatory shear index responses to single-leg heat stress and moderate whole body heat stress. Leg blood flow (*A*) and oscillatory shear index (*B*) in the CFA during single leg heat stress and moderate whole body heat stress (T_c_ + 1°C). Data are mean ± SE. *Significant change from baseline.

In both studies, mean shear rate significantly increased in all three arteries over the duration of passive heat stress (*P* < 0.05; Table 2). Increased shear in the CFA and SFA occurred as a result of an increased anterograde flow and shear rate component in conjunction with a significant decrease in opposing retrograde flow and shear. Mean PFA shear increased due to increases in anterograde shear rates only, as retrograde flow and shear remained unchanged throughout. The combination of an increased anterograde and decreased retrograde shear component in both the CFA and SFA led to significant decreases in the OSI. The PFA OSI was significantly lower than CFA and SFA at baseline but finished at levels similar to those of the other two vessels (final value: 0.08 ± 0.03). Exposure to moderate whole body heat stress (*study 1*) resulted in similar blood flows, shear rates, and OSIs in the CFA and PFA compared with isolated leg heat stress (Fig. 5), although SFA blood flow and OSI were significantly higher and lower, respectively (*P* < 0.05). Further severe heat stress resulted in additional increases in flow to all vessels alongside an almost complete abolition of oscillatory flow (maximum OSI = 0.05).

**Table 2.** Arterial diameters and flow profiles during whole body heat stress followed by single leg cooling and isolated single leg heat stress

	Whole body Heat Stress					Isolated Leg Heat Stress	
	Baseline	Mild	Moderate	Intense	Severe	Baseline	End
Diameter, cm							
CFA	0.96 ± 0.04	0.95 ± 0.04	0.96 ± 0.04	0.96 ± 0.04	0.96 ± 0.04	0.83 ± 0.01	0.84 ± 0.02
SFA	0.75 ± 0.03	0.73 ± 0.03	0.76 ± 0.03	0.76 ± 0.03	0.76 ± 0.03	0.67 ± 0.02	0.68 ± 0.02
PFA	0.56 ± 0.03	0.57 ± 0.02	0.57 ± 0.02	0.58 ± 0.03	0.58 ± 0.03	0.61 ± 0.04	0.61 ± 0.04
*V*_mean_, cm/s							
CFA	6.6 ± 0.6	13.3 ± 2.1*	20.8 ± 1.7*	28.4 ± 2.1*	28.5 ± 2.4*	7.4 ± 0.4	22.6 ± 2.7*
SFA	6.1 ± 0.3	16.6 ± 2.1*	26.8 ± 1.9*	33.9 ± 2.7*	34.4 ± 2.8*	6.2 ± 0.6	22.6 ± 3.9*
PFA	6.2 ± 0.6	7.7 ± 1.0*	11.7 ± 0.8*	14.4 ± 1.2*	16.8 ± 1.8*	5.0 ± 0.6	13.6 ± 3.3*
*V*_ant_, cm/s							
CFA	10.5 ± 1.0	16.8 ± 1.9*	23.5 ± 0.9*	30.3 ± 1.2*	30.2 ± 1.3*	13.2 ± 0.8	25.3 ± 2.6*
SFA	10.1 ± 0.8	19.8 ± 1.8*	28.9 ± 0.8*	35.1 ± 1.4*	36.1 ± 1.6*	12.7 ± 1.1	25.9 ± 3.9*
PFA	7.3 ± 0.7	9.0 ± 1.1*	12.6 ± 0.9*	15.5 ± 1.5*	18.7 ± 1.7*	9.7 ± 1.0	17.0 ± 2.8*
*V*_ret_, cm/s							
CFA	3.6 ± 0.5	3.1 ± 0.6	2.7 ± 0.6	0.5 ± 0.7*	0.5 ± 0.4*	5.9 ± 0.8	2.8 ± 0.6*
SFA	3.9 ± 0.7	2.3 ± 0.3*	1.4 ± 0.2*	0.1 ± 0.6*	0.3 ± 0.3*	6.5 ± 0.7	3.2 ± 0.8*
PFA	1.1 ± 0.2	1.0 ± 0.3	1.3 ± 0.2	1.1 ± 0.3	1.1 ± 0.4	2.5 ± 0.5	1.8 ± 0.2
SR_mean_, s^−1^							
CFA	28.9 ± 3.7	59.8 ± 12.2*	88.5 ± 6.6*	126.0 ± 11.9*	126.3 ± 11.0*	34.9 ± 1.9	107.1 ± 14.5*
SFA	33.1 ± 2.4	96.4 ± 12.2*	143.4 ± 5.0*	185.5 ± 16.0*	187.4 ± 14.6*	37.8 ± 3.7	138.9 ± 28.8*
PFA	44.0 ± 5.6	56.9 ± 10.4*	81.6 ± 7.9*	101.3 ± 11.7*	124.1 ± 15.2*	35.7 ± 5.5	98.4 ± 25.0*
SR_ant_, s^−1^							
CFA	44.0 ± 5.8	72.4 ± 10.7*	99.6 ± 5.7*	128.1 ± 10.4*	128.2 ± 11.0*	62.9 ± 4.3	120.1 ± 14.3*
SFA	53.7 ± 5.6	108.5 ± 12.9*	150.6 ± 5.0*	185.9 ± 13.9*	188.9 ± 14.7*	77.6 ± 6.7	159.4 ± 28.6*
PFA	52.0 ± 6.2	64.1 ± 9.3*	91.1 ± 8.2*	109.2 ± 12.2*	132.7 ± 16.3*	66.7 ± 7.1	120.7 ± 22.1*
SR_ret_, s^−1^							
CFA	15.1 ± 2.6	12.7 ± 2.1	11.2 ± 2.3*	2.1 ± 2.9*	1.9 ± 1.6*	28.0 ± 4.1	13.0 ± 2.8*
SFA	20.6 ± 4.1	12.1 ± 1.7*	7.2 ± 0.8*	0.5 ± 3.1*	1.5 ± 1.5*	39.8 ± 4.3	19.5 ± 4.7*
PFA	8.0 ± 1.7	7.1 ± 1.9	9.5 ± 1.9	7.9 ± 2.6	8.6 ± 2.8	18.5 ± 4.7	13.0 ± 2.3
OSI							
CFA	0.25 ± 0.02	0.16 ± 0.04*	0.10 ± 0.02*	0.02 ± 0.02*	0.01 ± 0.01	0.30 ± 0.02	0.10 ± 0.02*
SFA	0.27 ± 0.02	0.10 ± 0.02*	0.05 ± 0.01*	0.01 ± 0.02*	0.01 ± 0.01	0.34 ± 0.01	0.12 ± 0.03*
PFA	0.13 ± 0.03	0.12 ± 0.04	0.09 ± 0.02	0.07 ± 0.02*	0.06 ± 0.02*	0.21 ± 0.03	0.10 ± 0.01*

Values are means ± SE for 8 participants. *V*_mean_, time-averaged mean velocity; *V*_ant_, time-averaged anterograde velocity; *V*_ret_, time-average retrograde velocity; SR_mean_, mean shear rate; SR_ant_, anterograde shear rate; SR_ret_, retrograde shear rate; OSI, oscillatory shear index.

* *P* < 0.05, significantly different from previous condition.

### Relationship Between Hemodynamic Responses and Temperatures During Whole Body and Isolated Limb Heat Stress

**Fig. 6.**
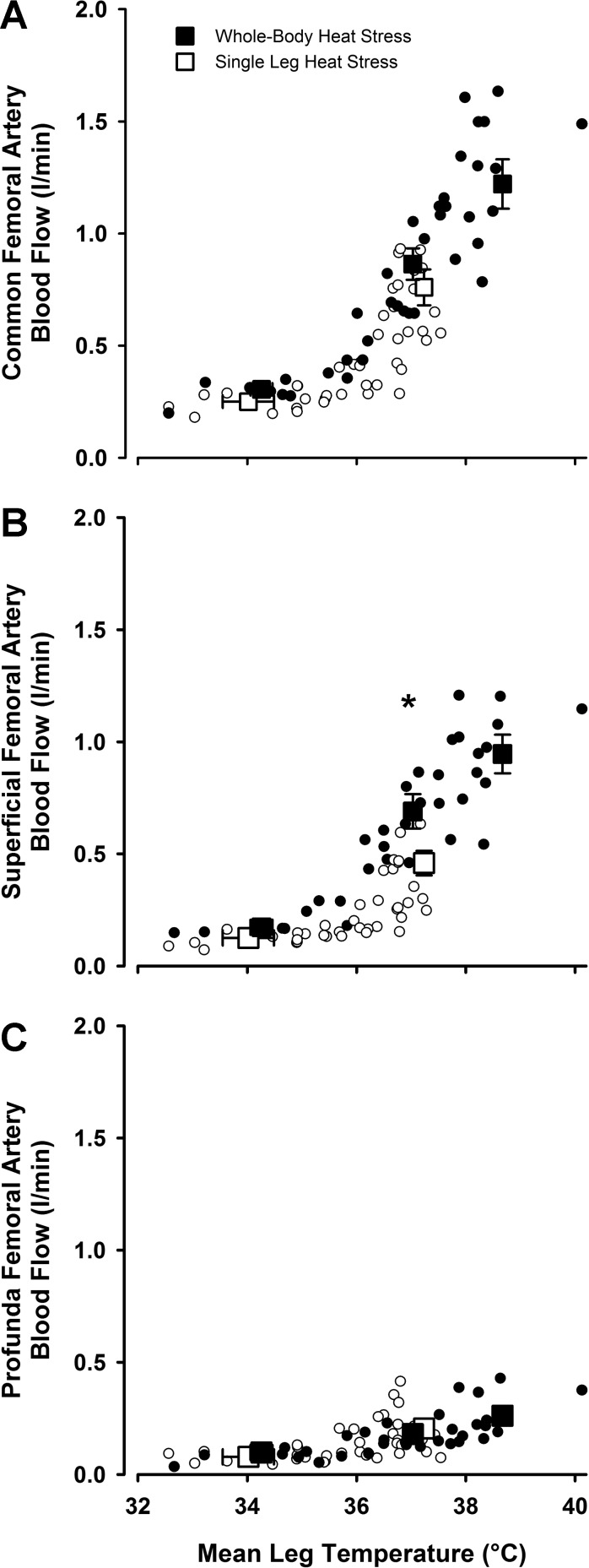
Arterial blood flows vs. mean leg temperature during passive whole body and single leg heat stress. Data are represented as mean ± SE (large black squares) for baseline (thermoneutral), moderate (T_c_ + 1°C) and severe (T_c_ + 2°C) whole body heat stress. Single leg baseline and heat stress values are also displayed (large white squares). *A*: CFA. *B*: SFA. *C*: PFA. *Significantly higher than equivalent single leg heat stress value. Individual data points are also displayed (small circles); *n* = 8 for whole body heat stress and *n* = 7 for single leg heat stress.

Whole limb blood flow was closely related to T̄_leg_ throughout both whole body and isolated leg heat stress (*R*^2^ = 0.77 and 0.56, respectively; *P* < 0.01; slopes: 0.19 ± 0.05 and 0.26 ± 0.04 l·min^−1^·°C^−1^; Fig. 6). During whole body heat stress, CFA (+0.93 l/min), SFA (+ 0.73 l/min), CFV (+ 0.71 l/min), and GSV (+ 0.23 l/min) all increased with increasing T̄_leg_, T̄_sk_, and T_c_ from baseline to conditions of intense heat stress (T_c_ + 1.5°C). Thereafter, no significant increases in flow were seen in any of these vessels, despite further increases in T_c_ and T̄_sk_ of 0.5 and 0.4°C, respectively, during severe heat stress. PFA displayed a modest yet steady increase throughout the entire heat stress protocol (+ 0.16 l/min). Isolated limb cooling maintained intense whole body heat stress (T_c_ + 1.6°C) while simultaneously lowering T̄_sk_ and T_sc_ to values similar to those seen at baseline (28.0 ± 1.6 vs. 32.4 ± 0.6°C and 31.5 ± 1.2 vs. 33.5 ± 0.7°C for T̄_sk_ and T_sc,_ respectively; *P* > 0.05). Modest decreases in flow were observed in the CFA (−0.24 l/min), SFA (−0.19 l/min), PFA (−0.08 l/min), and CFV (−0.19 l/min; *P* < 0.05 for all) in parallel to the decline in T̄_leg_ but not in the GSV (*P* > 0.05; Fig. 4).

**Fig. 7.**
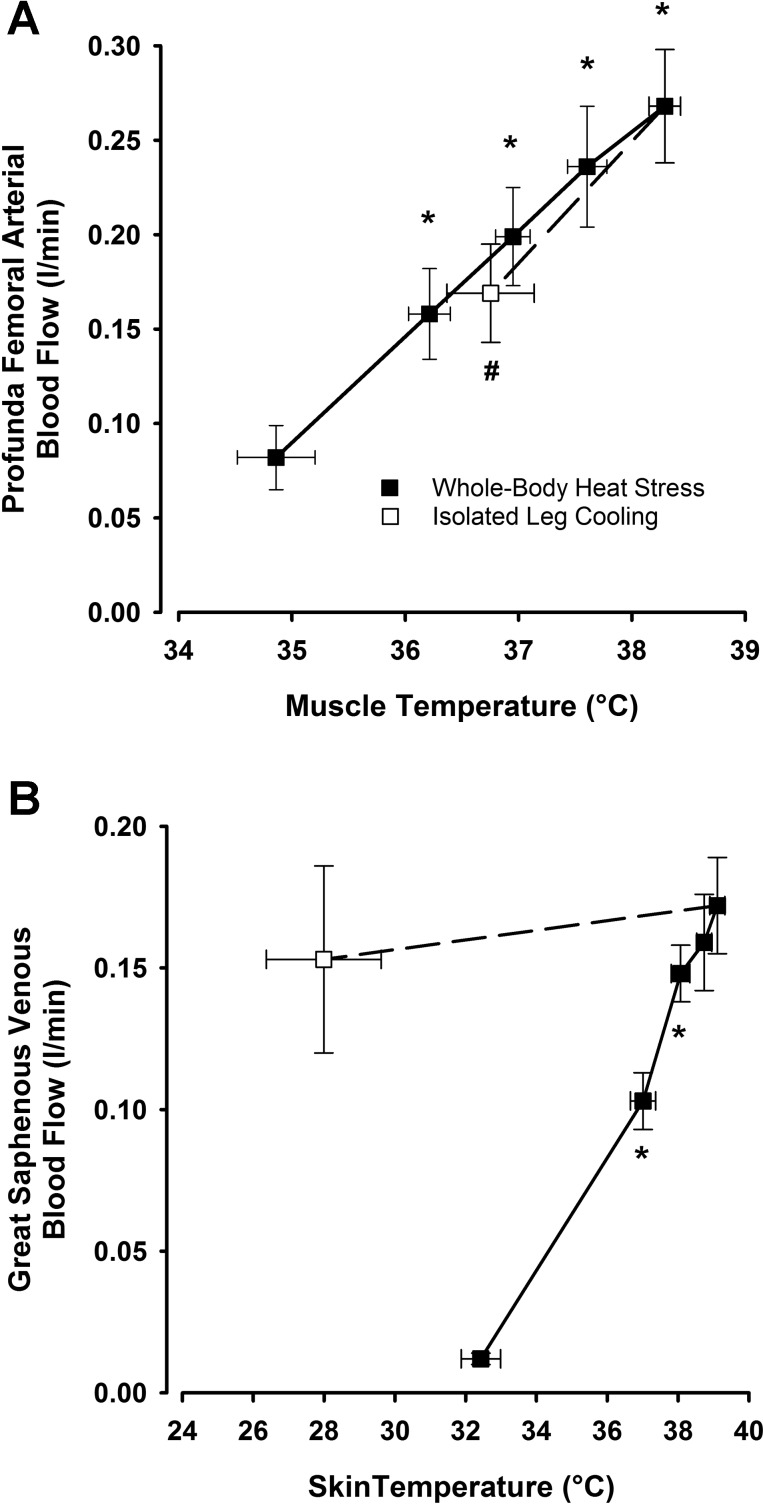
Relationships between local blood flow and temperatures during isolated single leg cooling following whole body heat stress. *A*: relationship between profunda femoral arterial flow (predominantly supplying deep tissues of the thigh) and local deep tissue temperature. Data are mean ± SE for 8 subjects. *B*: relationship between GSV blood flow (predominantly draining superficial tissues of the thigh) and local subcutaneous temperature during both severe whole body passive heat stress and subsequent local thigh cooling with arterial cuff occlusion. *Significantly higher than previous measurement. #Significantly lower than severe heat stress.

To investigate the relationship between different hemodynamic responses within the leg and their respective tissue temperatures, additional blood flow measurements were made in the GSV and PFA during leg cooling following an arterial occlusion at the level of the knee (Fig. 7). Blood flow through the GSV (major drainage vessel of the skin and subcutaneous tissues of the thigh) was closely related to increases in surrounding T_sk_ throughout all stages of passive heat stress (*R*^2^ = 0.81; *P* < 0.001), with rapid increases in both responses being observed at the start of the protocol before a subsequent plateau in both when approaching severe heat stress. This relationship was lost when leg T̄_sk_ and T_sc_ were rapidly cooled under conditions of high T_c_, with GSV flow remaining virtually unaffected despite decreases in leg T̄_sk_ and T_sc_ to levels lower than that recorded at baseline (25.9 ± 2.0 and 30.3 ± 1.3°C, respectively; *P* < 0.05). In contrast, blood flow through the PFA (main arterial supply to the deep tissues of the thigh) was closely related to both increases in local T_m_ during isolated leg heat stress (*R*^2^ = 0.59; *P* < 0.001) and the subsequent decreases in T_m_ observed during isolated leg cooling.

## DISCUSSION

This study sought to characterize hemodynamic adjustments within the major arteries and veins of the human leg during exposure to passive heat stress and attempted to elucidate the relative contribution of local vs. core temperatures in leg blood flow distribution via the manipulation of different regional temperatures. Both single leg and intense whole body heat stress led to progressive increases in blood flow and shear rates supplying the three major arteries of the leg alongside the virtual abolition of oscillatory shear profiles within the vessels. While further elevations in heat stress to severe levels were not accompanied by any further increases in flow to the CFA or SFA, continued modest increases to the PFA occurred in line with rising muscle temperature. Isolated single leg heat stress provided equivalent increases in leg blood flow and improvements in OSI compared with moderate levels of whole body heat stress but without the accompanying elevations in T_c_ and cardiovascular strain. Blood flow in the GSV (major vessel draining the skin) and CFV (major vessel draining the entire leg) also increased progressively with the rise in both T_c_ and T̄_leg_, with the deep venous system accounting not only for blood flow returning from the deep tissues of the leg but also a significant proportion of blood flow from the skin and superficial tissues. In line with previous studies from this laboratory (7, 29), the strikingly similar hemodynamic responses to both isolated leg and moderate whole body heat stress in the face of significantly different T_c_ suggest that limb tissue hyperemia with heat stress to moderate levels is closely coupled to local temperature. However, as heat stress progressed to more severe levels, rapid cooling of a single leg while maintaining intense levels of whole body hyperthermia resulted in a small decrease in flow draining the superficial tissues alone, suggesting that increased T_c_ has no effect on deep tissue perfusion at any level of heat stress.

### Leg Blood Flow Distribution and Shear Rate Profiles During Heat Stress

This study is the first to investigate the distribution of blood flow through each of the major arterial and venous vessels supplying and draining the leg from normothermic conditions to levels of skin and internal body hyperthermia approaching an individual's level of thermal tolerance. Arterial inflow to the leg increased in line with elevations in both T̄_leg_ and T_c_ to levels of intense heat stress (T_c_ + 1.5°C), thereafter plateauing with exposure to severe heat stress (Fig. 3*A*). While the majority (∼80%) of the increase in leg blood flow was directed through the SFA supplying the superficial tissues of the thigh and the lower leg, a threefold increase in blood flow through the PFA was also observed throughout the duration of the protocol. With blood flow through the PFA supplying the thigh alone [confirmed in the present study by an unchanged blood flow during leg cuff occlusion (Fig. 3*C*) and previously in anatomical studies showing that this vessel predominantly perfuses deep tissues; Ref. 21], these observations strongly suggest a linear increase in skeletal muscle blood flow in response to increasing levels of heat stress; agreeing with a number of recent studies (7, 13, 16, 29) and further challenging the dogma that increases in limb blood flow during internal body and skin hyperthermia are confined to the skin alone (9, 10, 32).

Simultaneous measurements of blood flow through the GSV and subsequent estimations of flow through the CFV shed light into the distribution of blood flow draining the major venous vessels of the leg. The GSV is often described as the major drainage vessel of the skin circulation, but previous studies utilizing moderate levels of heat stress have suggested that up to 50% of the increase in skin blood flow is drained through the deep venous system (1), most likely via the multiple perforating vessels linking the superficial and deep venous veins. Findings from this study appear to support this notion, with only 40% of the 0.5 l/min increase in thigh blood flow (during leg occlusion) returning directly through the GSV. Expanding upon the prior study investigating leg venous return during moderate heat stress (1), we observed that further increases in whole body heat stress to intense levels (T_c_ + 1.5°C) led to further substantial increases in leg blood flow that were matched through increases in CFV blood flow alone, as GSV blood flow remained essentially unchanged (Fig. 4). Together, these observations suggest that isolated leg heating and whole body heating to moderate level of heat stress have the potential to increase venous blood flow to an equal extent in both the superficial and deep venous systems of the leg, but that any further increases in leg blood flow during more severe levels of heat stress are diverted to the deep venous system for return to the heart. With the deep venous system being particularly at risk of thrombotic events in certain patient populations where lower leg movement is restricted (2), these findings reveal the ability of passively induced hyperthermia to substantially increase deep venous flow independently of lower limb exercise, although whether this can be applied chronically to reduce thrombotic risk remains to be determined.

Another aim of this study was to characterize the blood flow profiles within the leg's major vessels to assess whether the present leg and whole body heat stress interventions enhance anti-atherogenic shear responses. Interestingly, we found that severe whole body heat stress led to a three- to fivefold increase in blood flow and mean shear rates and the complete abolition of oscillatory shear profiles within each of the major arteries of the leg (Table 2). While the increased cardiovascular strain associated with heat stress has the potential to be detrimental to both health and exercise capacity, the idea has previously been advanced that whole body or local heat stress may be an effective nonpharmacological means for improving cardiovascular health in certain clinical populations (6, 14, 19), with part of these improvements attributed to enhancements in vascular endothelial health resulting from increased anterograde and decreased retrograde shear rates (37, 39–41). The majority of studies to date looking at repeated heat exposure as a method of “thermal conditioning” have employed indirect or whole body heat stress to moderate levels (6, 14, 19). Here, we report that moderate whole body heat stress to T_c_ + 1°C has little additional benefit to leg blood flow and shear rate patterns in any of the major vessels supplying and draining the leg compared with those observed during isolated leg heat stress, with no significant difference observed in any variable in the CFA and PFA and only modest improvements in blood flow and OSI values recorded in the SFA. These findings show for the first time that localized limb heat stress has the potential to increase blood flow and anti-atherogenic shear rates in the major arterial vessels of the leg to levels equivalent to those seen during moderate whole body heat stress, although without the added cardiac strain (increase in heart rate of up to 20–30 beats/min and cardiac output of up to 2 l/min) and thermal discomfort associated with whole body heat stress protocols. Whether the magnitude of changes in flow profiles observed is sufficient to cause chronic endothelial adaptation in the leg and whether the same benefits are observed in elderly/clinical populations compared with the healthy study group reported here, however, remain to be investigated.

### Temperature and Regulation of Leg Perfusion with Passive Heat Stress

The increases in leg blood flow (up to 0.9 l/min) and vascular conductance were closely related to local limb tissue temperatures (*R*^2^ = 0.77 and 0.56 for whole body and isolated leg heat stress, respectively; *P* < 0.01). These occurred despite noticeable differences in T_c_ and Q̇ between the two studies of 0.6°C and 2.1 l/min, respectively, agreeing with previous work from this laboratory indicating that local temperature-sensitive mechanisms account for the vast majority of increases in limb skin and deep tissue blood flow during whole body direct heat stress to moderate levels of core hyperthermia (7). This raises the question as to whether local temperature and/or temperature-sensitive mechanisms is/are also a critical regulatory factor when T_c_ increases further during intense and severe heat stress. Although the precise regulatory mechanisms of blood flow regulation were not tested in the current study, a number of observations support a critical role for local temperature-sensitive mechanisms during whole body heat stress to levels approaching those of an individual's thermal tolerance. Firstly, the slopes relating leg blood flow to local temperatures (T̄_leg_) during both isolated limb heat stress and all whole body heat stress conditions were comparable (0.26 ± 0.04 vs. 0.19 ± 0.05 l·min^−1^·°C^−1^; respectively, *P* = 0.32; Fig. 5), indicating that additional increases in T_c_ do not appear to alter the flow-to-temperature relationship during even severe levels of heat stress, and therefore suggesting a continued important role for local temperature in limb blood flow regulation under these conditions. These findings agree with recent research from this (7, 29) and other (13) laboratories but disagree with a number of classical studies in which the majority of limb vasomotor response to internal hyperthermia was attributed to central reflex mechanism (3, 11, 33). This fundamental difference in the contribution of central vs. local mechanisms, however, can most likely be attributed to the different methodological approaches employed to raise body temperature, with the use of indirect passive heating (11, 33) or lower body exercise (3) in previous studies resulting in increased T_c_ but relatively little increase in skin and deep tissue temperatures at the site of measurement in the forearm. This contrasts with the direct whole body heating approach in the current study, in which direct heating of the site of measurement resulted in rapid elevations of T_sk_ to ∼39°C, followed by gradual increases in T_m_ and T_c_ to 38.3 and 38.8°C, respectively, once a positive skin-to-subcutaneous tissue temperature gradient had been established. A second observation was that, despite a steady increase in T_c_ during whole body heat stress, the time course of increased tissue perfusion within the upper and lower portions of the leg in the current study differed in line with a differing increase in local tissue temperatures. In short, the presence of a rapid and sustained increase in T_thigh_ to ∼40°C during the early stages of whole body heating was accompanied by the attainment of a plateau in thigh blood flow during levels of moderate heat stress, despite T_c_ only being elevated by 1°C. In contrast, lower leg blood flow at the same core temperature remained relatively low in line with a slower warming of local temperatures at this site (∼36°C during moderate heat stress), with the majority of blood flow increases in this portion of the leg occurring between moderate and intense levels of T_c_ alongside further increases in both T_c_ and T_calf_. Taken together, these observations suggest that under the current form of heat stress, the direct heating of the site of measurement (both during isolated and whole body heat stress) results in local temperature-sensitive mechanisms accounting for the majority of the elevation in leg blood flow before T_c_ rises sufficiently to exert any significant vasodilatory effects through central temperature mediated reflexes.

To further investigate this hypothesis, we employed the use of two additional interventions following the attainment of severe heat stress. On the one hand, we employed a rapid single limb cooling protocol to quickly lower local temperatures while maintaining an overriding high T_c_, while on the other hand, we occluded blood flow at the level of the knee and assessed the relationship between *1*) T_sk_ and GSV flow (representing relative changes in skin tissue perfusion alone), and *2*) T_m_ and PFA flow (major supplier to deep tissues of the thigh and taken to predominantly represent relative changes to skeletal muscle tissues alone). Rapid cooling of the leg following heat stress had no significant effects on GSV blood flow (Δ −19 ml/min; *P* > 0.05; Fig. 7*B*), despite drastic decreases in local T_sk_ to levels below those recorded at baseline (final value of ∼28°C compared with 33°C at baseline). These findings agree with previous heat stress studies where an overriding cutaneous vasodilation was found to be maintained during cooling from prior heat stress (5, 42, 45), presumably due to a continued vasodilatory response mediated through the sympathetic nervous system. A novel observation of this study was that in contrast to superficial tissue blood flow, deep tissue blood flow in the thigh following an occlusion at the level of the knee appeared to continue to be mediated solely at a local level during all stages of heat stress (Fig. 6*A*), with PFA blood flow not only increasing in a linear fashion with T_m_ to the limits of thermal tolerance but also decreasing at the same rate when local muscle tissues were rapidly cooled. This finding expands upon previous research showing the local control of skeletal muscle blood flow during moderate heat stress (13) by showing that this response is maintained throughout severe levels of heat stress (despite an accompanying plateau in skin blood flow) and can be reversed with local cooling even when T_c_ is significantly elevated. Although the molecular mechanisms underpinning these changes in flow distribution were not investigated in this study, there are a number of potential candidates that may be responsible. Significant increases in skin blood flow during heat stress is a well-documented phenomenon and has been shown to be mediated through multiple synergistic and redundant pathways that include, but are not limited to, central-sympathetic drive, axon-reflex responses, and a large nitric oxide component (17, 18, 23). In contrast to the cutaneous circulation, pathways responsible for the moderate increases in muscle blood flow are not as well documented but may involve temperature-dependent erythrocyte ATP release (15), shear-mediated nitric oxide release (28), and/or actions mediated through transient receptor potential vanilloid (TRPV) channels (12).

### Experimental Considerations

There are a number of experimental considerations that need to be taken into account when interpreting the current findings. Firstly, the Modelflow technique for the estimation of Q̇ has previously been reported to underestimate increases in systemic blood flow during heat stress conditions when compared with invasive thermodilution techniques (38), and as such, Q̇ values reported here are lower than expected. If we assume a stable stroke volume response across all levels of heat stress as reported in most of the literature (8, 24, 35), the increase in Q̇ would amount to 4.9 l/min, rather than 3.3 l/min estimated by the Modelflow technique. This limitation has no bearing on findings of this study, however, as the primary outcome of changes in leg blood flow were measured using duplex-Doppler ultrasound. Secondly, it should be noted that different individuals were tested between *study 1* and *study 2*, making this a between-subjects design rather than the gold standard within-subjects design. That said, we are confident that this study design remains robust due to *1*) the careful selection of age- and sex-matched participants for *study 2*, and *2*) a wealth of data from this laboratory over the years showing highly reproducible leg blood flows with this method of heat stress during both local and whole body heating (∼ 0.3 l·min^−1^·°C_muscle_^−1^) (7, 29, unpublished data). This reproducibility is also supported in the current study by the individual data points displayed in Fig. 6.

### Conclusions

Passive whole body heat stress that includes direct heating of the legs leads to substantial increases in blood flow and shear rates to all major arteries and veins supplying and draining the leg alongside a virtual abolition of oscillatory shear profiles within the vessels. The increase in skin blood flow during moderate to severe heat stress appears to be drained predominantly through the deep venous systems of the leg, with blood flow through the GSV plateauing before this point. These changes in limb tissue blood flow are predominantly mediated through local mechanisms, resulting in similar hemodynamic responses during both isolated limb and moderate whole body heat stress. As heat stress progresses to more severe levels, elevated core temperatures affect skin but not skeletal muscle circulations, as evidenced by the close association between local temperature changes and muscle, but not skin, blood flows when core temperatures are elevated. These findings further our knowledge of leg hemodynamic responses during heat stress and provide evidence of potentially beneficial vascular alterations during isolated limb heat stress that are equivalent to those experienced during exposure to moderate levels of whole body hyperthermia. Previous research in the human forearm has shown that repeated increases in blood flow and shear rates within the brachial artery result in chronic beneficial adaptations to the vasculature (6, 25). Further studies using validated measures of subclinical endothelial function and vascular health, alongside studies elucidating mechanistic responses, are required to establish whether these hemodynamic changes have the potential to exert a chronic beneficial effect in a variety of patient populations.

## DISCLOSURES

No conflicts of interest, financial or otherwise, are declared by the author(s).

## AUTHOR CONTRIBUTIONS

Author contributions: S.T.C. and J.G.-A. conception and design of research; S.T.C., S.J.T., and J.G.-A. performed experiments; S.T.C. analyzed data; S.T.C., S.J.T., and J.G.-A. interpreted results of experiments; S.T.C. prepared figures; S.T.C. drafted manuscript; S.T.C., S.J.T., and J.G.-A. edited and revised manuscript; S.T.C., S.J.T., and J.G.-A. approved final version of manuscript.
